# Comparative analysis of serum lipid profiles in patients with and without gallstones: A prospective cross-sectional study

**DOI:** 10.1016/j.amsu.2019.04.003

**Published:** 2019-04-24

**Authors:** Sikandar Hayat, Zarbakht Hassan, Shabbar Hussain Changazi, Anam Zahra, Muhammad Noman, Muhammad Zain ul Abdin, Haris Javed, Armghan Haider Ans

**Affiliations:** a Services Institute of Medical Sciences Lahore, Pakistan; b Fatima Memorial Hospital Lahore, Pakistan; c Sahara Medical College Narowal, Pakistan

**Keywords:** Gallstone disease, Serum lipid profile, Comparison

## Abstract

**Objectives:**

Gallbladder disease is one of the most common diseases of the gastrointestinal tract. Various studies have shown an association between gallstones and an alteration in the serum lipids. The objective of this study was to compare serum lipid profile of gallstone patients with the controls.

**Methods:**

This prospective cross-sectional study was conducted in the Surgical Department of the Services Institute of Medical Sciences from August 2017 to August 2018. A total of 50 patients were included in the study after screening through the inclusion criteria. A control group of 50 inpatients with no personal or family history of gallstones were also recruited for comparison. Results were expressed as mean with standard deviation. Students t-test was used to compare the data between the patients and the control groups (p < 0.05 was considered statistically significant). SPSS software, version 20 was used for statistical analysis.

**Results:**

The mean age of the patients was 40.90 years and that of controls was 34.74 years. 46 patients were females and 44 controls were females. The serum cholesterol levels were high in the patients as compared to the controls but the comparison was not statistically significant. Serum triglycerides levels were high in the patients as compared to the controls and the analysis was statistically significant. Furthermore, the serum HDL levels were low in the patients as compared to the controls with a statistically significant p-value. However, the serum LDL levels were low in the patients as compared to the control group.

**Conclusion:**

It was concluded that serum triglyceride levels and serum HDL levels were statistically significant in gallstone patients and there was a positive correlation between these parameters and gallstone disease.

## Highlights

• Gallbladder disease is one of the most common diseases of the gastrointestinal tract in Pakistan.
• Various studies have shown an association between gallstones and an alteration in the serum lipids.
• This study was conducted to find out the association of dyslipidemias with formation of gallstones.
• Serum lipid profile of gallstones patient was compared with inpatient control group.
• It was concluded in study that serum triglyceride levels and serum HDL levels were statistically significant in gallstone patients and there was a positive correlation between these parameters and gallstone disease.

## 1 Introduction

Gallstone disease is a common disorder of the gastrointestinal tract, having a prevalence of 10%–15% and an incidence of 1.4% per year in the adult population of developed countries [1,2]. Women are more common victims of the gall stone disease as compared to men [3].

Gallstones are classified into three main types: cholesterol, pigment, and mixed gallstones. Cholesterol gallstones contain 51%–99% of pure cholesterol. Mixed gallstones have cholesterol plus calcium salts, bile acids, phospholipids and bile pigments. In about 70–80% of the cases, gallstones are mixed stones [4]. The process of gall stone formation is multiplex. Major factors that govern the stone formation are: super saturation of the secreted bile, concentration of bile inside the gall bladder, crystal triglyceride nucleation, and abnormal gall bladder emptying [5]. Cholesterol super saturation of the bile is the most crucial factor [6,7]. Cholesterol is insoluble in water, it is secreted from the canalicular membrane in unilamellar phospholipid vesicle. Cholesterol solubility in the bile requires sufficient bile salts and phospholipids, predominantly phosphatidylcholine. If there is an excess of cholesterol or reduced phospholipids and/or bile acid, multi lamellar vesicles are formed causing nucleation of the cholesterol crystals which leads to the stone formation. The secretion of cholesterol supersaturated lithogenic bile, decreased concentration of phospholipids, gallbladder dysmotility, delayed large bowel transit times (favoring reabsorption of deoxy-cholic acid), and the resection of ileum (depleting the acid pool) have all been implicated in the gallstone formation [8].

Hyperlipidemia is generally characterized by high serum levels of total cholesterol, triglycerides, low density lipoproteins (LDL), and low levels of high-density lipoprotein (HDL). There are controversies that hyperlipidemias are associated with the gallstones. Some studies have showed a significant association of hyperlipidemias with gallstones especially hypertriglyceridemia and increased LDL levels [3,[8], [9], [10]] while others showed no significant association between hyperlipidemias and gallstones [1,11,12]. Therefore, the aim of this study was to compare the lipid abnormalities in the patients presenting with gallstones with the controls.

## 2 Materials & methods

This prospective cross-sectional study was conducted in the surgical department of the Services Institute of Medical Sciences from August 2017 to August 2018. A total of 50 patients were included in the study after screening through the inclusion criteria. 50 inpatients with no history of gallstones were recruited as control group. Male or female patients with the gall stone disease having age from 20 to 70 years were included in this study. Gallstones were diagnosed through the ultrasound as mobile echoes in the gallbladder with acoustic shadows. Patients with acalculous gallbladder disease on ultrasound, terminal ileal resection, hemolytic diseases (hereditary spherocytosis, sickle cell anemia on history and CBC film), liver cirrhosis (on abdominal ultrasound) and patients on antihyperlipidemic drugs were excluded from the study. An approval was taken from the ethical committee of the hospital. Written informed consent was taken from every patient. Blood samples drawn from the patients and controls were analyzed for serum cholesterol, serum triglycerides, serum LDLs and serum HDL levels. All the results were recorded on a performa. Results were expressed as mean with standard deviation. Students t-test was used to compare the data between the patients and the control groups (p < 0.05 was considered statistically significant). SPSS software, version 20 was used for statistical analysis. The study was registered in clinical trial.gov with ID number NCT03804775. All the work performed in this study was in line with the STROCSS criteria [13].

## 3 Results

A total 100 people were recruited in the study, out of which 50 were patients and 50 were controls. The mean age of the patients was 40.90 years and that of controls was 34.74 years. In the study, 46 patients were female and 44 controls were female. The study evinced that the serum cholesterol levels were high in the patients as compared to the controls but the comparison was not statistically significant. Serum triglyceride levels were high in the patients as compared to the controls and the analysis was statistically significant. Furthermore, it was concluded from the study that serum HDL levels were low in the patients as compared to the controls with a statistically significant p-value. However, the study depicted that serum LDL levels were raised in the controls as compared to the patients. The mean serum cholesterol, triglyceride, LDL, HDL were shown below (Table 1).

**Table 1:** Showing means of lipid profile in disease and controls.

Lipid Parameters	Disease Means (SD)	Control group Means (SD)	P value
Cholesterol	184.60 (37.65)	181.08 (33.97)	0.625
Triglyceride	198.12 (48.40)	171.98 (54.57)	0.013
LDL	118.40 (23.96)	122.12 (35.92)	0.544
HDL	29.54 (8.406)	40.34 (11.61)	0.000

## 4 Discussion

In the study, the mean age of the patients was 40.90 ± 12.65 years. Similar results were also seen in the studies conducted by Gul H et al. [14] and Weerakoon HT et al. [12] with the mean ages of 43.18 ± 13.970 years and 44.6 ± 10.4 years respectively. However, in the study performed by Öner C et al. [11] illustrated that mean age of the patients was 52.6 ± 13.07 years.

In this study, the majority of the patients were female gender having percentage of 92%. Studies done by Weerakoon HT et al. [12], Gul H et al. [13]and Halgaonkar P et al. [15] also showed that the majority of the patients belonged to the female gender showing hormonal role in pathogenesis of gallstones.

The mean serum cholesterol levels were high in the patient as compared to the control group however, the correlation of high cholesterol levels and gallstones was not statistically significant. Similarly, Channa NA et al. [9] and described that the serum cholesterol levels were not statistically significant in the gallstones patients as compared to the control group. Similar results were also demonstrated by Öner C et al. [11] in their study. In contrast to the above results, Al-Saadi N et al. [16] found that the serum cholesterol levels were significantly elevated in the gallstones patients as compared to the control group. Although the saturation of bile with cholesterol has definite role in pathogenesis of gallstones but association of gallstones and high level of serum cholesterol levels in patients is controversial in literature and can be explained by multiple factors like genetics, geographical, social and dietary habits in pathogenesis of different type of gallstones.

This study depicted that the mean serum triglyceride levels of the patients were significantly raised as compared to the control group. This result was in concordance with studies conducted by Al-Saadi N et al. [16]. Similar results were also illustrated by Bell GD et al. [17]. On the contrary, some investigators elaborated that there was no significant association of serum triglyceride in gallstone patients and control groups [1,11,14]. There is some evidence that triglyceride in bile decreases gallbladder motility but correlation of serum triglyceride levels with gallstone formation is debatable in literature search.

In the present study, mean serum HDL levels were significantly low in the patients as compared to the control group. Similar results were also found by studies conducted by Batajoo H et al. [1] and Chen et al. [18]. On the other hand, some studies showed no association between low HDL levels and gallstone disease [11,16]. The main source of biliary cholesterol is HDL cholesterol but studies have shown that low HDL cholesterol has positive correlation with gallstones. Still some studies revealed no association of low HDL and gallstone pathogenesis.

In the study, mean serum LDL level in the patients was low as compared to the control groups but results were not statistically significant. Study carried out by Al-Saadi N et al. [16] showed that LDL concentration had no significant difference compared to the control. These results were in contrary with the other studies that demonstrated positive correlation between serum LDL levels and gallstones patients [1,14]. In literature, there were studies describing positive correlation of serum LDL levels and gallstones and others showing no association however, in this study the serum LDL levels were lower in patients as compared to control group.

## 5 Conclusions

It was concluded that serum triglyceride levels and serum HDL levels were statistically significant in the gallstone patients as compared to the control group and there was positive correlation between these parameters and the gallstone disease.

## Provenance and peer review

Not commissioned, externally peer reviewed.

## Ethical approval

Ethical Approval was taken from Institute Review Board of Services Institute of Medical Sciences and will be provided on request.

## Sources of funding

Nil.

## Author contribution

Sikandar Hayat: data collection, literature search, study design.

Zarbakht Hassan: data collection, literature search, study design.

Shabbar Hussain Changazi: study design, writing, data analysis.

Anam Zahra: study design, writing, data analysis.

Muhammad Noman: critical review, final layout.

Muhammad Zain ul Abdin: critical review, final layout.

Haris Javed; data analysis.

Armghan Haider Ans: critical review.

## Conflicts of interest

Nil.

## Research registration number

In clinical trial.gov NCT03804775.

## Guarantor

Shabbar Hussain Changazi.

## Appendix A Supplementary data

the_strocss_statement_checklist.docx the_strocss_statement_checklist.docx
